# Multifeature Extraction and Seafloor Classification Combining LiDAR and MBES Data around Yuanzhi Island in the South China Sea

**DOI:** 10.3390/s18113828

**Published:** 2018-11-08

**Authors:** Mingwei Wang, Ziyin Wu, Fanlin Yang, Yue Ma, Xiao Hua Wang, Dineng Zhao

**Affiliations:** 1 College of Geomatics, Shandong University of Science and Technology, Qingdao 266590, China; ericking1992@foxmail.com; 2 Key Laboratory of Submarine Geosciences, Second Institute of Oceanography, Hangzhou 310012, China; zhaodineng@sio.org.cn; 3 School of Electronic Information, Wuhan University, Wuhan 430079, China; mayue19860103@163.com; 4 Sino Australian Research Centre for Coastal Management, University of New South Wales, Canberra, BC 2610, Australia; x.h.wang@unsw.edu.au; 5 State Key Laboratory of Satellite Ocean Environment Dynamics, Second Institute of Oceanography, Hangzhou 310012, China

**Keywords:** LiDAR, MBES, multifeature, seafloor classification, SVM

## Abstract

Airborne light detection and ranging (LiDAR) full waveforms and multibeam echo sounding (MBES) backscatter data contain rich information about seafloor features and are important data sources representing seafloor topography and geomorphology. Currently, to classify seafloor types using MBES, curve features are extracted from backscatter angle responses or grayscale, and texture features are extracted from backscatter images based on gray level co-occurrence matrix (GLCM). To classify seafloor types using LiDAR, waveform features are extracted from bottom returns. This paper comprehensively considers the features of both LiDAR waveforms and MBES backscatter images that include the eight feature factors of the LiDAR full waveforms (amplitude, peak location, full width half maximum (FWHM), skewness, kurtosis, area, distance, and cross-section) and the eight feature factors of MBES backscatter images (mean, standard deviation (STD), entropy, homogeneity, contrast, angular second moment (ASM), correlation, and dissimilarity). Based on a support vector machine (SVM) algorithm with different kernel functions and penalty factors, a new seafloor classification method that merges multiple features is proposed for a beneficial exploration of acousto-optic fusion. The experimental results of the seafloor classification around Yuanzhi Island in the South China Sea indicate that, when LiDAR waveform features are merged (using an Optech Aquarius system) with MBES backscatter image features (using a Sonic 2024) to classify three types of sands, reefs, and rocks, the overall accuracy is improved to 96.71%, and the kappa reaches 0.94. After merging multiple features, the classification accuracies of the SVM, genetic algorithm SVM (GA-SVM) and particle swarm optimization SVM (PSO-SVM) increase by an average of 9.06%, 3.60%, and 2.75%, respectively.

## 1. Introduction

Seafloor features reveal important marine environment information for the fields of marine geological surveying, marine engineering construction, and mineral resources on the seabed. To survey seafloor features, the traditional direct sampling method and indirect remote sensing methods that use acoustics, optics, and electromagnetic waves are normally used. Based on the pulse signal obtained by airborne bathymetric light detection and ranging (LiDAR) and the backscatter intensity obtained by multibeam echo sounding (MBES), the return waveforms and mosaic sonar images, respectively, can be generated. The shapes of LiDAR waveforms and the grayscale and texture features of sonar images vary with seafloor type for different surface profiles and reflectivity.

A full laser waveform contains both the sea surface and bottom peaks [1,2]. Through waveform processing, airborne bathymetric LiDAR can not only acquire the depth of shallow water areas but also distinguish different seafloor types from bottom returns. Cottin et al. proposed a method to classify shallow seabed textures and algae coverage types by extracting waveform parameters of bottom returns collected by the bathymetric LiDAR of scanning hydrographic operational airborne LiDAR survey (SHOALS). The overall accuracy of this classification was up to 67% [3]. Narayanan et al. input waveform parameters of the Optech’s SHOALS-3000 hydrographic mapping system to a decision tree and rotation forest algorithms for classification, and the average overall accuracy was 91% [4]. Lin et al. used Gaussian and Weibull functions to fit and extract waveform parameters, which were combined with artificial identification data to classify the types of ground objects. The classification accuracies were approximately 78% and 83% for the support vector machine (SVM) and random forest algorithms, respectively [5].

The MBES system can simultaneously acquire bathymetric and backscatter data. These data are usually used to explain seabed geology and sedimentary processes, identify geotechnical disasters and leakage of sea bottom gases, and assess environmental impacts [6,7]. The methods that use MBES data for seafloor classification are primarily based on SVM, learning vector quantization (LVQ), self-organizing feature map (SOM) classifiers, and cluster analysis methods [8,9]. Tang et al. used a Simrad EM3000 multibeam sounder (Simrad Yachting, Oslo, Norway) to collect backscatter data and in situ sediment sampling data from Jiaozhou Bay in Qingdao, China, then used the back propagation neural network (BPNN) and genetic algorithm optimization of the BPNN (GA-BPNN) for classification, and the accuracy was up to 80.2% and 85.8%, respectively [10]. Giovanni et al. studied the relationships between bathymetric data, backscatter data, angle response curve and sediment particle size, and seaweed distribution. They calculated the relative scattering intensity thresholds of five kinds of sediment (gravelly sands, sandy gravels, slightly gravelly muddy sands, Posidonia oceanica on sediments, and Posidonia oceanica on hardgrounds), but did not provide the classification results [11]. Li et al. compared 14 classification combinations of machine learning methods using MBES data. It was found that classification of the random forest combined with a general kriging algorithm was the best and could reduce the prediction error by 17% [9]. Lark et al. constructed an image texture for classification based on a co-kriging algorithm. The results were validated by the sediment types sampled in the experimental area, and the prediction accuracy was as high as 70% [12].

Using bathymetry data collected by bathymetric LiDAR, Sun et al. proposed a hybrid K-means and SVM algorithm (KSVM) and calculated the gray level co-occurrence matrix (GLCM) to classify K-means as clusters [13]. The results indicated that, compared with the traditional SVM algorithm, KSVM increased the overall accuracy by 24% and the kappa coefficient by 0.31. Selvarajan et al. used the SVM algorithm to classify point cloud data and aerial images collected by Riegl LMS-Q680. In their method, a total of six extracted features were considered: three LiDAR features (elevation, echo intensity, and return number) and three image features (intensity value in the red, green, and blue bands). The results showed that classification accuracy based on LiDAR features was 86% and based on image features was 65%, while overall accuracy of the fusion reached 88% [14]. Zhang et al. applied a standard deviation (STD) based method to quantitatively characterize terrain complexity of the Yuanzhi Island surveys. In their method, disturbance of terrain irregularities was accounted for by robust estimation, and a depth calibration procedure was introduced to eliminate the disturbance of the depth-dependent noise component. The results showed that the presented method provided better characterization of seafloor terrain complexity. Meanwhile, the obtained surface roughness (SR) index was found to be significantly correlated with in situ coral abundance observations, which suggested that the SR index was a suitable indicator of habitat complexity in a coral reef environment [15].

In summary, there are several methods of seafloor classification, based on return waveform features of laser pulses, based on backscatter image features of MBES, and based on multisource data and multiple features. However, the current classification methods mostly use airborne LiDAR and MBES data separately. For the classification algorithms that merge the multifeatures of both LiDAR and MBES data, overall accuracy is less than 90% when the type of seafloor around islands or reefs contains more than three species. This paper is intended to ascertain more seafloor information through the merging of features that are extracted by both LiDAR and MBES data. Three kinds of SVM classifiers (genetic algorithm (GA) and particle swarm optimization (PSO)) are used and trained, thus achieving improved classification accuracy for seafloor classification in shallow water areas.

## 2. Multifeature Extraction Algorithm

### 2.1. Feature Extraction of LiDAR Return Waveforms

The Gaussian decomposition method is mainly used to process waveforms of small footprint full-waveform airborne LiDAR systems. First, it is assumed that the emitted laser pulse is an approximately Gaussian distribution. It is then assumed that the target return signal is also a Gaussian distribution, so it can be approximately considered as a superposition of several Gaussian components. Therefore, the Gaussian function is used for waveform fitting. The return waveform can be described by Equation (1) as follows:

(1) $$y=f\left(t\right)=N_{b}+\sum_{i=1}^{n}A_{i}exp\left[-{\left(t-b_{i}\right)}^{2}/2\sigma_{i}^{2}\right],$$

where *N_b_* indicates the background noise of the original waveform, *n* indicates the number of Gaussian components, *A_i_* represents the peak of the *i*th Gaussian component, *b_i_* represents the peak position of the *i*th Gaussian component, *σ_i_* indicates the full width at half maximum (FWHM) of the *i*th Gaussian component, and *y* is the amplitude of the waveform at time *t* [16,17].

**Figure 1:**
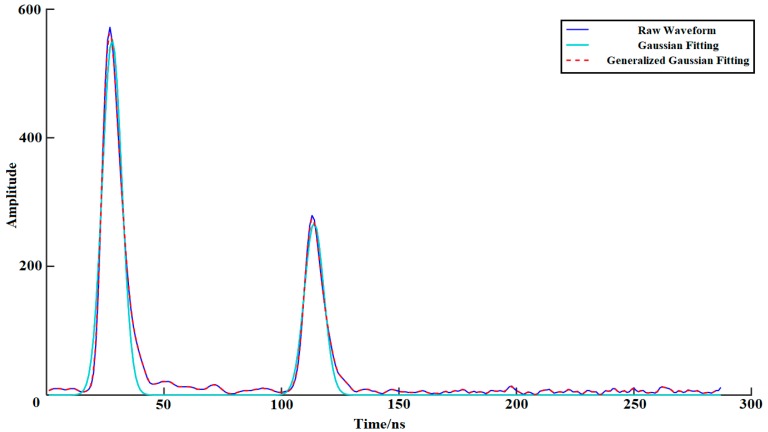
Schematic of Gaussian fitting: the dark blue curve represents the raw waveform, the blue curve represents the Gaussian fitting, and the red dotted line represents the generalized Gaussian fitting.

For small footprint airborne LiDAR systems, the signal distribution often does not meet the standard Gaussian distribution. As shown in Figure 1, this paper aims to use a more complex Gaussian function model to improve the waveform fitting accuracy and extract more information from the original signal. For symmetric waveforms, we can use standard Gaussian functions for fitting. If the detected peak is asymmetric, then a lognormal function can be used for fitting. However, for some complicated symmetric waveforms with deformations, we consider the use of generalized Gaussian functions for fitting [18]. The expressions of these three fitting functions are as follows:

(2) $$f_{G,j}\left(x\right)=a_{j}exp\left(-\frac{{\left(x-\mu_{j}\right)}^{2}}{2\sigma_{j}^{2}}\right),$$

(3) $$\;f_{L,j}\left(x\right)=a_{j}exp\left(-\frac{{\left(ln\left(x-s_{j}\right)-\mu_{j}\right)}^{2}}{2\sigma_{j}^{2}}\right),\;$$

(4) $$\;f_{GG,j}\left(x\right)=a_{j}exp\left(-\frac{{\left|x-\mu_{j}\right|}^{\alpha_{j}^{2}}}{2\sigma_{j}^{2}}\right).\;$$

The eight features of a LiDAR return waveform are: amplitude, peak location, FWHM, skewness, kurtosis, area, distance, and cross-section [19]. Expressions and explanations of LiDAR features are given in Table 1.

### 2.2. Feature Extraction of MBES Backscatter Images

The return intensity is a complex physical quantity that is related to various factors, such as emission frequency, seafloor type, and grazing angle. Different seafloor types may have different textures on the acoustic images. Texture directly reflects the roughness of the seabed surface, which can be used for classification. Co-occurrence matrix statistical analysis is the most widely used method for texture analysis. A rectangular window is generally taken from the same cluster on the sonar image to calculate the co-occurrence matrix, and then the co-occurrence matrix is statistically analyzed [20,21].

GLCM evaluates the relationship between pixels at a certain distance along a certain direction (0°, 45°, 90° and 135°). The value of the matrix element (*i*, *j*) is equal to the probability of appearing, *p*, or the frequency of the pair of pixels with gray levels *i* and *j*.

The eight features of MBES backscatter images are: mean, standard deviation (STD), entropy, homogeneity, contrast, angular second moment (ASM), correlation, and dissimilarity. Expressions and explanations of MBES features are given in Table 2.

## 3. Seafloor Classification Methods

### 3.1. Classification Model Construction

For seafloor classification and identification applications, SVM has some advantages over other methods. If the parameters are properly selected, the classification accuracy is relatively higher. Therefore, it has great value in the application of seafloor classification. However, the disadvantage of this method is that the choice of parameters is difficult to determine and is more sensitive to the classification results and accuracy. To solve these problems, this paper not only performs traditional SVM classification but also uses two optimization algorithms (GA-SVM and PSO-SVM) to classify the samples of selected seafloor features. The selection of kernel function parameters and penalty factors has a great influence on the accuracy of the SVM. Therefore, the penalty factor *c* and kernel function parameter *g* are optimized by the algorithm, which achieves better prediction classification accuracy.

#### 3.1.1. SVM Algorithms

The principle of SVM is to use the optimization tool to find the optimal separating hyperplane (OSH) in a high-dimensional vector space. Therefore, two categories can be divided through this plane. The support vector machine in the transformation space can be written as follows:

(5) $$\;f\left(X\right)=sgn\left(\overset{N}{\underset{i=1}{\sum }}y_{i}a_{i}^{*}K\left(X_{i}\cdot X_{j}\right)+b^{*}\right).\;$$

In Equation (5), *K* is a kernel function; the most common kernel functions are the polynomial function, the radial basis function (RBF), and the sigmoid function (Equation (6)):

(6) $$\;\{\begin{array}{c} \mathrm{polynomial}:K\left(X_{i}\cdot X_{j}\right)={\left(g\cdot X_{i}{}^{T}X_{j}+g\right)}^{d},\;g>0\\ \mathrm{RBF}:K\left(X_{i}\cdot X_{j}\right)=exp\left(-g\cdot \|X_{i}-X_{j}\|{}^{2}\right),\;g>0\\ \mathrm{sigmoid}:\;K\left(X_{i}\cdot X_{j}\right)=tanh\left(g\cdot X_{i}{}^{T}X_{j}+g\right) \end{array},\;$$

where *g* is the kernel function parameter and RBF is the radial basis function. In Equation (5), the coefficient is the solution to the following optimization problem:

(7) $$\;\max H\left(a\right)=\overset{N}{\underset{i=1}{\sum }}a_{i}-\frac{1}{2}\overset{N}{\underset{i=1}{\sum }}\overset{N}{\underset{j=1}{\sum }}y_{i}y_{j}a_{i}a_{j}K\left(X_{i}\cdot X_{j}\right).\;$$

b* can be obtained by satisfying the following support vector samples:

(8) $$\;y_{i}\cdot \left(\overset{N}{\underset{i=1}{\sum }}y_{i}a_{i}^{*}K\left(X_{i}\cdot X_{j}\right)+b^{*}\right)-1=0.\;$$

The objective function for optimization is as follows:

(9) $$\;\begin{array}{c} \min\frac{1}{2}\omega^{T}\omega +c\overset{l}{\underset{i=1}{\sum }}\xi_{i},\\ s.t.\;\;y_{i}\left(\omega^{T}\phi \left(x_{i}\right)+b\right)\geq 1-\xi_{i},\;\left(\xi_{i}\geq 0,\;i=1,\ldots ,l\right),\; \end{array}\;$$

where *ξ_i_* is the hinge loss, and *c* is the penalty factor.

#### 3.1.2. Improved SVM Algorithm Based on GA

The GA is a search algorithm used to solve optimization in computational mathematics and is a type of evolutionary algorithm [22,23]. First, the population is initialized, and the initial population of individuals is randomly generated; the individual gene strands in the population are decoded into the corresponding kernel function numbers, kernel function parameters, and error penalty factors; these three parameters are substituted into the SVM to train and test the training samples and the testing samples. Second, the selection operator is executed according to the principle of optimal preservation and worst substitution. Based on the fitness ratio selection strategy, the selection probability *p_i_* for each individual *i* is as follows:

(10) $$\;p_{i}=\frac{f_{i}}{\sum_{j=1}^{N}f_{i}}.\;$$

In Equation (10), *f_i_* = *k*/*F_i_*, *F_i_* is the fitness value for each individual *i*, and since it is better that the fitness value is smaller, its reciprocal will be calculated before the individual is selected. *k* is the coefficient, and *N* is the number of individuals. Then, the crossover operation is executed. Since the individual uses real-coded, the cross-operation method uses the real cross method. The *k*th and *l*th chromosomes in the *j*-bit operation are as follows:

(11) $$\;\{\begin{array}{c} a_{kj}=a_{kj}\cdot \left(1-b\right)+a_{lj}b\\ a_{lj}=a_{lj}\cdot \left(1-b\right)+a_{kj}b \end{array},\;$$

where *b* is a random number between 0 and 1.

Finally, the mutation operator is executed and the *j*th gene *a_ij_* of the *i*th individual is selected to mutate. The mutation method is as follows:

(12) $$\;a_{ij}=\{\begin{array}{c} a_{ij}+\left(a_{ij}-a_{max}\right)\cdot f\left(\lambda \right),\;r>0.5\\ a_{ij}+\left(a_{min}-a_{ij}\right)\cdot f\left(\lambda \right),\;r\leq 0.5 \end{array},\;$$

where *a_max_* is the upper bound of gene *a_ij_*, *a_min_* is the lower bound of gene *a_ij_*, *f*(*λ*)= *r*_2_⋅(1 − *λ*/*G_max_*)^2^, *r*_2_ is a random number, *λ* is the number of the current iteration, G*_max_* is the maximum number of evolutions, and *r* is a random number in the range of [0, 1].

When GA is used for SVM parameter optimization, real number-based encoding is used. The penalty factor *c* and the kernel function parameter *g* for the original SVM give a wide search range. Within this preset range, each possible *c* and *g* will be converted to specific chromosomes that can be accepted by the GA to complete the conversion from the feasibility solution space to the chromosome search processing space.

#### 3.1.3. Improved SVM Algorithm Based on PSO

PSO is another swarm intelligence-based optimization algorithm, along with the ant colony algorithm (ACA), in the field of computational intelligence. PSO is a type of evolutionary computing technology that is based on swarm intelligence. Compared with GA, PSO has no selection, crossover, or mutation operations. Instead, it conducts research by using particles in the solution space to follow the best examples [24]. A Each particle’s fitness function *f*(*x*) is defined by Equation (13). When *f*(*x*) is smaller, the adaptability is stronger:

(13) $$\;\mathrm{fitness}=\frac{1}{RMSE\left(\sigma^{2},\gamma \right)}.\;$$

The parameters of the particle swarm include the particle velocity and position. *n* particles are randomly generated in the *R^n^* space, and ***X_i_*** = [*x*_1_, *x*_2_, …, *x_n_*] and the velocity matrix ***V***(*t*) = [*v*_1_, *v*_2_, …, *v_n_*] are populated. The best fitness value *f*(*x_i_*) of each particle is compared with the optimal fitness value *f*(*g_besti,i_*) of all particles. If *f*(*x_i_*) < *f*(*g_besti,i_*), then the original global best fitness value is replaced with the best fitness value of the particle, and the current state of the particle is saved. Then, according to the improved PSO model:

(14) $$\;\{\begin{array}{c} v_{id}\left(t+1\right)=wv_{id}\left(t\right)+c_{1}rand\left\{\right\}\cdot \left[p_{id}-x_{id}\left(t\right)\right]+c_{2}rand\left\{\right\}\cdot \left[p_{gd}-x_{id}\left(t\right)\right]\\ x_{id}\left(t+1\right)=x_{id}\left(t\right)+v_{id}\left(t+1\right). \end{array}\;$$

The particle velocity and position are updated, and the new population ***X***_(*t*+1)_ is generated. Finally, the binary bit of the particle is updated and the terminating condition is checked. The terminating condition of the algorithm is either the maximum number of iterations or when the evaluation value is less than the given accuracy, whichever is least.

When PSO is used for the optimization of SVM parameters, particles can be represented as ***X_i_*** = [*c_i_*, *g_i_*], and particle velocity can be expressed as *v_i_* = [*v_ci_*, *v_gi_*]. The goal of using PSO to optimize SVM parameters is to maximize the classification accuracy of the SVM algorithm, so that the maximum classification accuracy of the SVM algorithm in the training data set is used as the fitness function in the PSO. Equation (14) is calculated iteratively to find the optimal SVM parameters c and g.

### 3.2. Data Processing and Classification Framework

#### 3.2.1. Classification Framework

**Figure 2:**
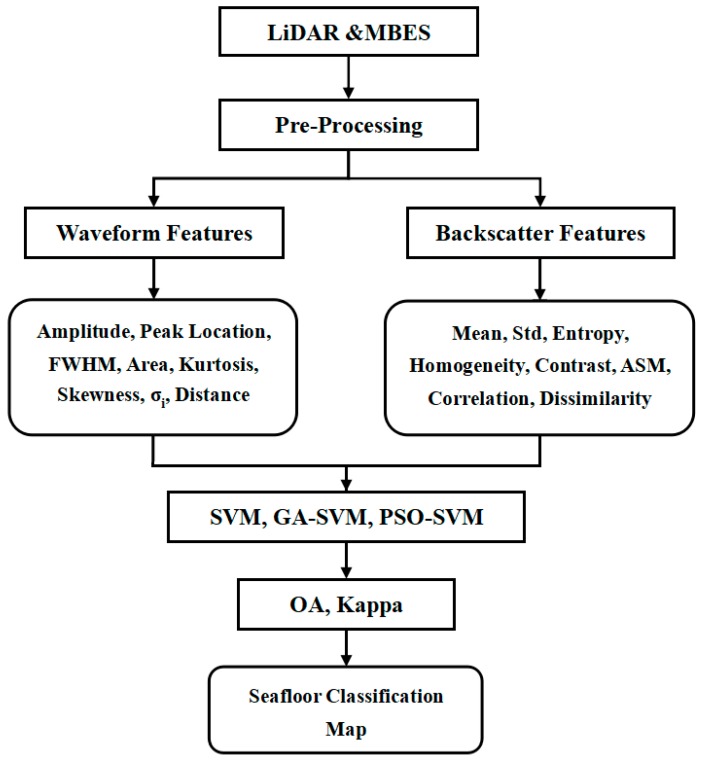
Framework of the proposed classification methods.

Figure 2 shows the framework of the proposed classification methods. After preprocessing, the waveform features and backscatter features are extracted from the LiDAR and MBES data. Then, these multifeatures (including the eight LiDAR features and eight MBES features) are assigned to three classifiers (SVM, GA-SVM and PSO-SVM) as training and testing samples. Thus, the classification accuracy of the nine combinations (LiDAR + SVM, LiDAR + GA-SVM, LiDAR + PSO-SVM, MBES + SVM, MBES + GA-SVM, MBES + PSO-SVM, LiDAR and MBES + SVM, LiDAR and MBES + GA-SVM, LiDAR and MBES + PSO-SVM) are evaluated as the overall accuracy (OA) and the kappa coefficient. Finally, the seafloor classification map is generated as experimental results.

#### 3.2.2. Classification Accuracy

There are some accuracy and error metrics listed below (a–d) to reflect the quality of the seafloor classification in the surveying area [25,26,27].

a. Producer’s accuracy and omission error

Producer’s accuracy (PA) is the map accuracy from the point of view of the map maker (the producer). This is how often real features on the ground are correctly shown on the classified map or the probability that a certain land cover of an area on the ground is classified as such. Producer’s accuracy is a complement of omission error (OE): PA = 100% − OE. It is also the number of reference sites classified accurately divided by the total number of reference sites for that class:

(15) $${\mathrm{PA}}_{i}=\frac{N_{ii}}{\sum_{j=1}^{n}N_{ij}}\times 100\%,$$

(16) $${\mathrm{OE}}_{i}=1-{\mathrm{PA}}_{i}.$$

b. User’s accuracy and commission error

User’s accuracy (UA) is accuracy from the point of view of a map user, not the map maker. This is how often a class on the map will actually be present on the ground. User’s accuracy is a complement of commission error (CE): UA = 100% − CE. It is calculated by taking the total number of correct classifications for a particular class and dividing it by the row total:

(17) $${\mathrm{UA}}_{j}=\frac{N_{jj}}{\sum_{i=1}^{n}N_{ij}}\times 100\%,$$

(18) $${\mathrm{CE}}_{j}=1-{\mathrm{UA}}_{j}.$$

c. Overall accuracy

Overall accuracy (OA) essentially tells us, out of all reference sites, what proportion were mapped correctly. Overall accuracy is usually expressed as a percent, with 100% accuracy being a perfect classification where all reference sites were classified correctly. Overall accuracy is the easiest to calculate and understand, but ultimately only provides the map user and producer with basic accuracy information:

(19) $$\mathrm{OA}=\frac{\sum_{i=k}^{n}N_{ij}}{\sum_{i,j=1}^{n}N_{ij}}\times 100\%.$$

d. Kappa coefficient

The Kappa coefficient is generated from a statistical test to evaluate the accuracy of a classification. Kappa essentially evaluates how well the classification performed compared to just randomly assigning values, i.e., whether the classification did better than random. The kappa coefficient can range from –1 to 1. A value of 0 indicates that the classification is no better than a random classification. A negative number indicates that the classification is significantly worse than random. A value close to 1 indicates that the classification is significantly better than random:

(20) $$\mathrm{Kappa}=\frac{P_{O}-P_{C}}{1-P_{C}}\times 100\%,$$

where *N_ij_* represents the element of column *i* and row *j* in the error matrix, *P_O_* represents observed agreement, and *P_C_* represents chance agreement.

## 4. Experiments and Analysis

### 4.1. Surveying Area

**Figure 3:**
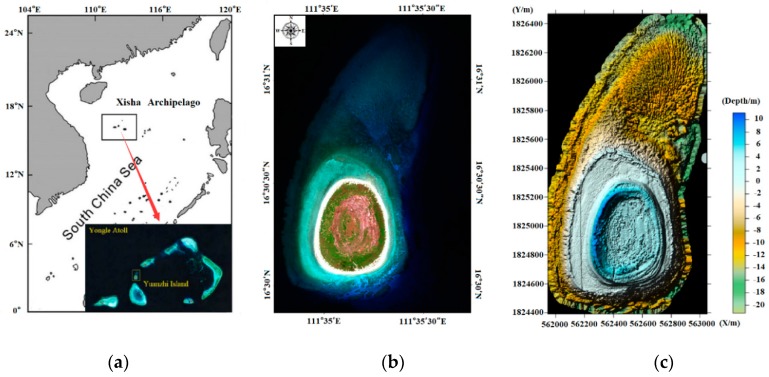
The survey area, Yuanzhi Island, is located in the Xisha Archipelago, South China Sea. (**a**) Location of the Xisha Archipelago; (**b**) overview map of the survey area; (**c**) 3D rendered water depth map of Yuanzhi Island.

The experimental area of this paper is located in Yuanzhi Island, Xisha Archipelago, South China Sea. The scope of the survey area is shown in Figure 3. The island is elliptical and is 700 m long from north to south, 500 m wide from east to west, and consists of an area of approximately 0.3 km^2^. This area has a typical tropical marine climate, where corals and plankton breed vigorously, forming a large number of coral reefs at the high platforms near the coast. At the same time, there are dense aquatic plants such as seaweed and kelp in the vicinity of the reef. Therefore, there are also many types of seafloor, including rocks, coral reefs, and sands, which makes this area suitable for detecting the effectiveness of different seafloor classification methods.

The airborne LiDAR in this paper uses the Optech Aquarius system (Optech Incorporated, Vaughan, ON, Canada). The system features a circular scanning mode, whose maximum bathymetric ability is 1.5 times the disk transparency (Secchi disc depth, SDD). The depth measuring accuracy is 0.25 m root mean square error (RMSE), which uses only a blue-green laser with a wavelength of 532 nm to achieve integrated over- and underwater surveying. The water in the survey area is clear and transparent, with an SDD of 9 m, an east–west width of 1.5 km, a north–south distance of 2 km, and a water depth of approximately 20 m. During data acquisition, the flying height is 300 m, the laser scanning nadir angle is 20°, the sounding frequency is 550 kHz, and the measuring point density is 5–10 points/m^2^. The MBES in this paper uses the SONIC 2024 system (R2Sonic, Austin, TX, USA). The underwater surveying is carried out in an airborne LiDAR survey area with a maximum range of 500 m, a span resolution of 1.25 cm, a coverage width of 10°–160°, 256 beams, and a measuring point density of 10–20 points/m^2^. There are no available MBES data in the nearshore area of Yuanzhi Island because the water is too shallow, and the boat cannot safely approach the survey area.

### 4.2. Experimental Data

**Figure 4:**
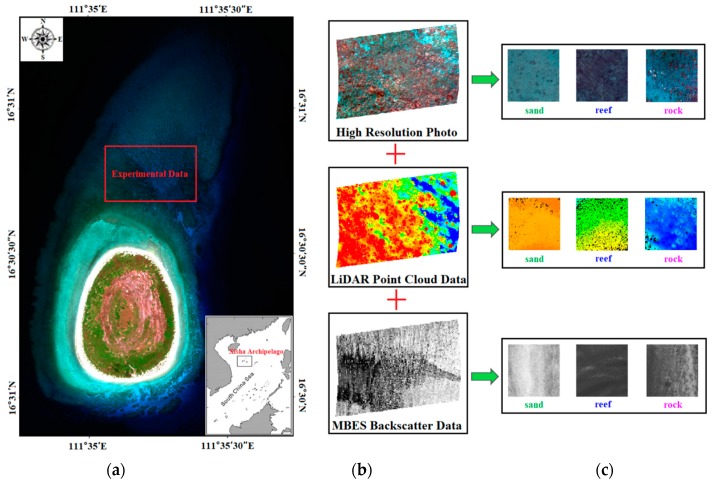
Location of survey area and experimental data. (**a**) Location of the whole island; (**b**) experimental data; (**c**) categories of seafloor.

**Figure 5:**
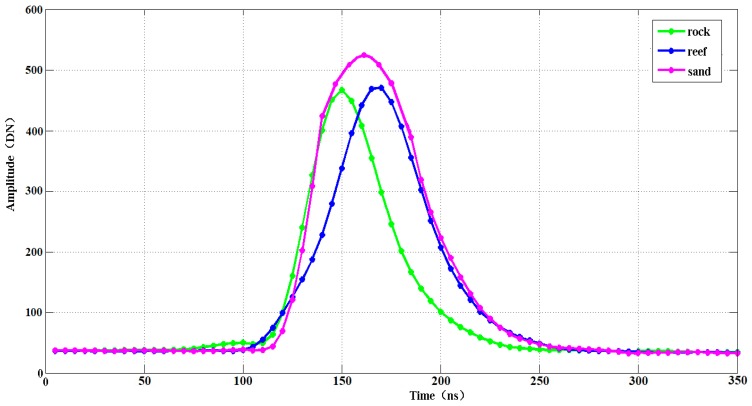
Different waveforms of the seafloor at the same time. Magenta waveform represents sand, blue waveform represents reef, and green waveform represents rock.

The experimental data in this paper include four primary components: LiDAR point clouds, LiDAR waveforms, MBES backscatter images and high-resolution images. According to an overview of the survey area and real high-resolution images, the seafloor can be divided into three types: sands, reefs, and rocks. Figure 4 shows the full view of the survey area and the specific location of the experimental data. Figure 5 shows the LiDAR bottom return waveforms of different seafloor types at the same time. Sample selection requires both real high-resolution images of the survey area and actual sampling data. Specifically, actual sampling data of the seafloor was acquired by an underwater video recorder (GoPro) in May 2016, thus in situ observations of the seafloor were collected and bathymetric data were used to designate sampling locations across the survey area. Constrained by the practical survey conditions, videos were collected at 28 different locations around the island. During the video sampling, the video recorder (tied to a plumb and a float ball) was dropped to the seafloor from the static ship. For stations within the MBES survey area, the horizontal positions of the ship were measured with a dual-frequency differential global positioning system (GPS) (centimeter accuracy). For stations in the shallower areas, the horizontal positions of the dingy were measured with a beacon-aided GPS, which provides horizontal positions with an accuracy of 1–2 m.

In this paper, we selected 1560 sand samples, of which 1092 were used for training. We also selected 518 reef samples, of which 362 were used for training. Finally, we selected 504 rock samples, of which 352 were used for training. The extracted sample feature information was input into SVM, GA-SVM, and PSO-SVM classifiers. A five-fold cross validation strategy was adopted and the classification results, including mean errors and accuracy metrics, are given in Section 4.3 [28,29,30].

### 4.3. Experimental Results

#### 4.3.1. Classification Results Based on LiDAR Waveform Features

**Figure 6:**
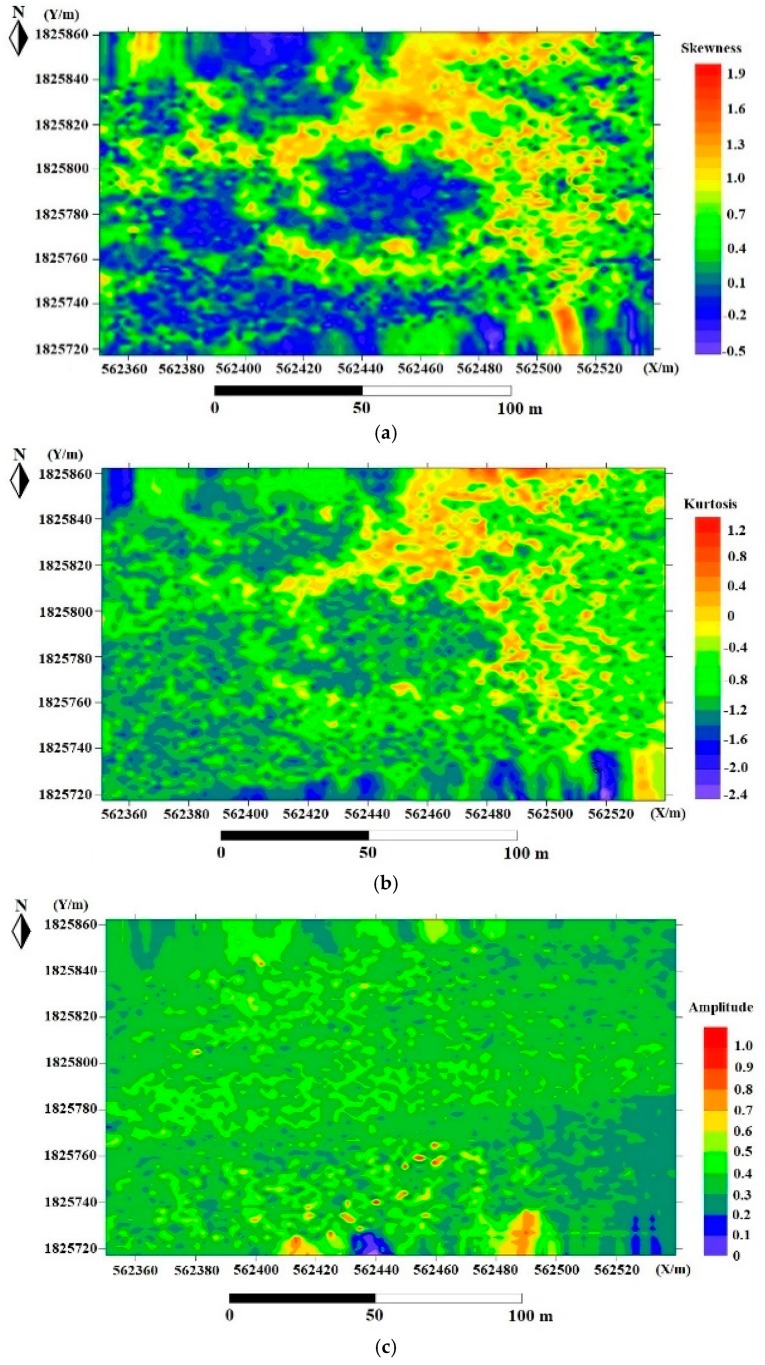

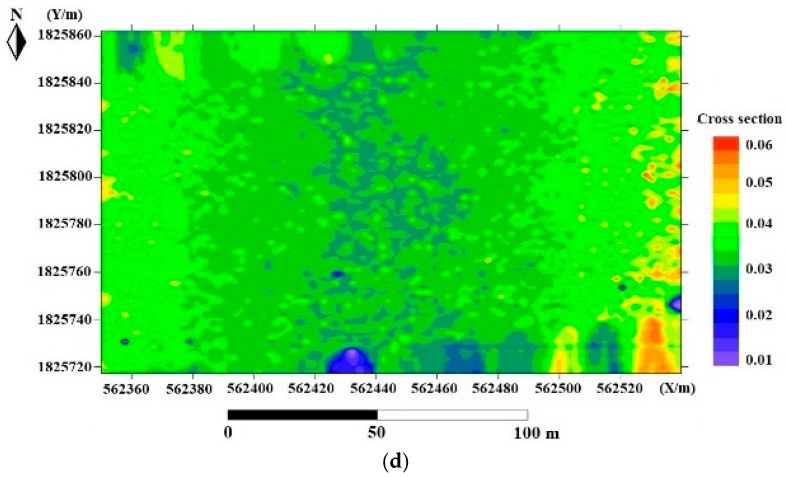
LiDAR waveform parameters: (**a**) skewness; (**b**) kurtosis; (**c**) amplitude; (**d**) cross-section. (Measuring point density is 10–20 points/m^2^, plane coordinate is WGS_1984_UTM_Zone_49N coordinate in meters).

The classification results based on LiDAR waveform features are shown in Table 3, which include the overall accuracy (OA), producer accuracy (PA), user accuracy (UA), and standard deviation (STD). Figure 6 shows the four LiDAR waveform features of skewness, kurtosis, amplitude, and cross-section. The model evaluation results are from five-fold cross validation.

#### 4.3.2. Classification Results Based on MBES Backscatter Features

The classification results based on MBES backscatter features are shown in Table 4, which include the overall accuracy (OA), producer accuracy (PA), user accuracy (UA), and standard deviation (STD). The model evaluation results are from five-fold cross validation.

#### 4.3.3. Classification Results Based on Multifeatures

**Figure 7:**
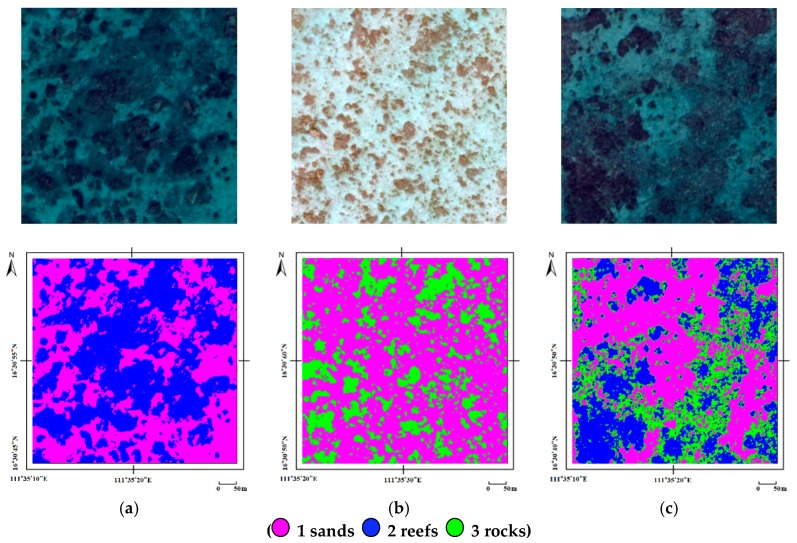
Classification results of different seafloors: (**a**) coverage area of sands and reefs; (**b**) coverage area of sands and rocks; (**c**) coverage area of sands, reefs, and rocks. Magenta represents sands, blue represents reefs, and green represents rocks.

The classification results based on multifeatures are presented in Table 5, which include the overall accuracy (OA), producer accuracy (PA), user accuracy (UA), and standard deviation (STD). Figure 7 shows the classification results of different seafloor coverage areas. The model evaluation results are from five-fold cross validation.

#### 4.3.4. Classification Results Analysis

**Figure 8:**
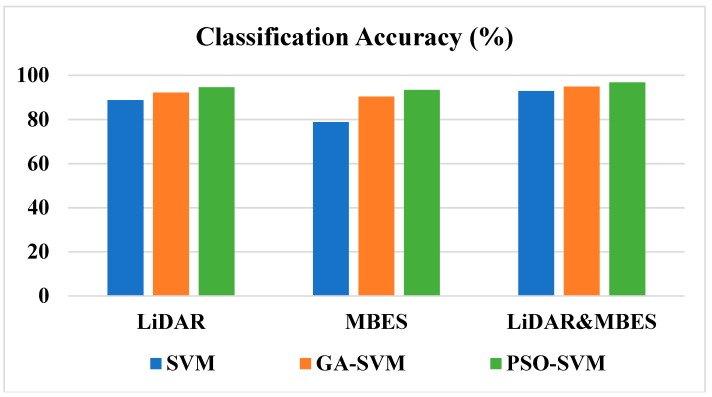
Statistics of the three combinations of classification accuracy. This is a comparison of the three algorithms (SVM, GA-SVM, PSO-SVM) before and after merging multifeatures.

The experimental results indicate that the classification accuracy significantly improved after merging the LiDAR and MBES multifeatures. After optimizing the parameters of the SVM classifier by using the GA and PSO algorithms, the classification accuracy also improved. Among the methods examined, the PSO-SVM classifier based on multifeatures had the highest overall accuracy of 96.71%, and its kappa coefficient was 0.94. The classification result using the SVM classifier based on MBES backscatter features had the lowest overall accuracy of 78.82% and a kappa coefficient of 0.61. The statistics of classification accuracy are illustrated in Figure 8, which shows the classification accuracy for the three algorithms (SVM, GA-SVM, and PSO-SVM) and the three models (LiDAR, MBES, and multifeatures). Compared to the accuracy of SVM, the accuracy of GA-SVM and PSO-SVM increased by an average of 5.62% and 8.08%. Figure 8 also indicates that after merging multifeatures of the LiDAR and MBES data, the classification accuracy of SVM improved by 4.10% and 14.02%, respectively, compared to that using the LiDAR or MBES features separately.

When classifying seafloor types based on LiDAR waveform features, the PSO-SVM classifier had the highest classification accuracy (up to 94.54%), and its kappa coefficient was 0.90. The SVM classifier had the lowest classification accuracy of 88.73% and a kappa coefficient of 0.80. Among the three types of seafloor, the classification accuracy of sands was relatively high. For the three classifiers, SVM, GA-SVM, and PSO-SVM, PA reached 96.86%, 97.31%, 97.82%, respectively. The classification accuracy of rocks was low. For SVM, GA-SVM, and PSO-SVM, PA was only 57.14%, 72.82%, and 83.33%, respectively. Through the above analysis, it was found that the performance of PSO-SVM was better than that of both SVM and GA-SVM.

**Figure 9:**
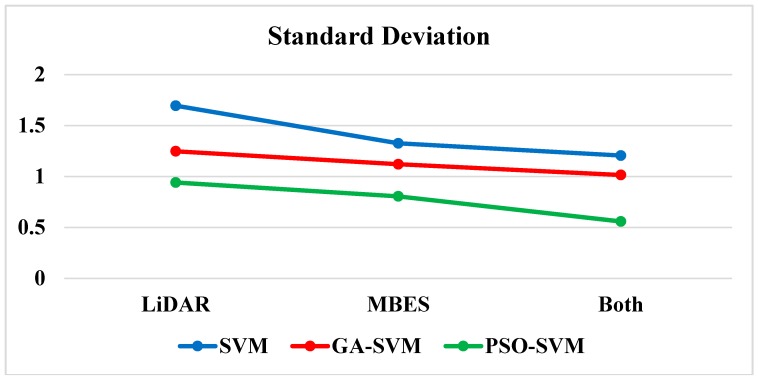
Standard deviations of different models based on five-fold cross validation.

**Figure 10:**
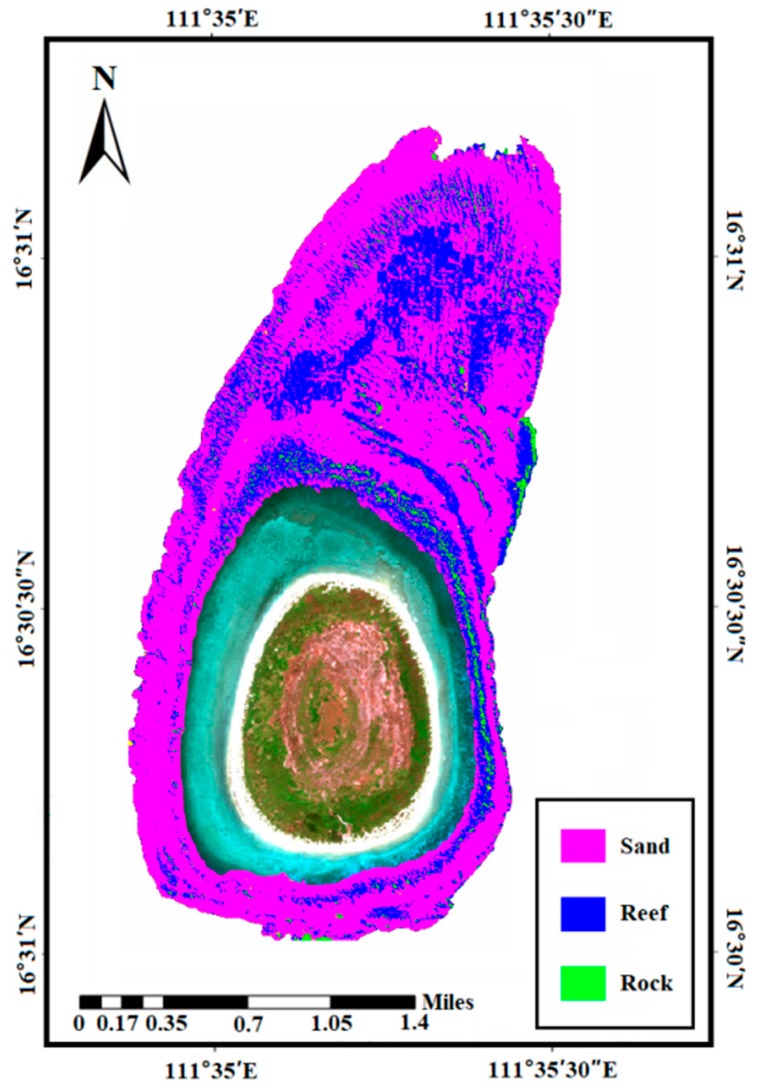
Overall classification results of the survey area based on seafloor.

When classifying seafloor types based on MBES backscatter features, the PSO-SVM classifier had the highest classification accuracy (up to 93.38%), and its kappa coefficient was 0.88. The SVM classifier had the lowest classification accuracy of 78.82% and a kappa coefficient of 0.61. Among the three types of seafloor, the classification accuracy of reefs was relatively high. For GA-SVM and PSO-SVM, PA reached 94.98% and 98.46%, respectively. However, the classification accuracy of rocks is relatively low. For the two classifiers, SVM and GA-SVM, PA reached 64.88% and 88.89%, respectively. Through the above analysis, it is found that the classification accuracy of PSO-SVM increased with the increase of particle swarm size. It can be seen from Figure 9 that the combination of the PSO-SVM classifier based on multifeatures had the highest classification accuracy and is thus the best method, and was applied to the whole survey area as shown in Figure 10. The data set was randomly shuffled and split into five groups. For each group, we used the group as a test data set, used the remaining groups as a training data set, and trained the model on the training set and evaluated it on the test set. The skill of the model was summarized using model evaluation scores from k runs. Through five-fold cross validation, the classification results (A–E) of the five groups were checked with the actual sampling data, and the standard deviations were also calculated (as shown in Table 6 and Figure 9).

As shown in Table 6 and Figure 9, it can be concluded that, after five-fold cross validation, the classification accuracy of the PSO-SVM is the highest among the three, and the standard deviation of the PSO-SVM is the lowest among the three.

Figure 10 shows the classification results when applying the best method (the PSO-SVM based on multifeatures) proposed in this paper to the whole survey area. Figure 10 is the classification result using multifeatures of the LiDAR and MBES data and the PSO-SVM classifier that corresponds to the best performance in Figure 8. In Figure 10, the results are essentially consistent with the actual terrain and seafloor distribution in the survey area. However, considering the computer performance, model fitting and other factors, our method may be affected by the number of samples. In general, classification efficiency is negatively correlated with the number of samples. The larger the sample size, the lower the classification efficiency.

## 5. Discussion

This study focused on providing an effective classification method for mapping the benthic substrate based on multifeatures extracted from LiDAR and MBES. According to the experimental results in Section 4, the following statements can be made.

(1) Data fusion

Registration is the process of transforming different data sets into one coordinate system. Data may come from different sensors, times, depths, or viewpoints. Although LiDAR and MBES measurements were collected individually, they were all transformed from a sensor coordinate system to the WGS-84 geodetic coordinate system and projected to the UTM plane coordinate system. During this process, measurement errors caused by mounting misalignment and attitude change were also considered. Thus, LiDAR and MBES data sets were accurately registered through coordinate transformation, map projection, and point matching.

(2) Sample size

Spatial resolution refers to the size or dimension of the smallest unit that can be distinguished in detail, which is used to characterize the product to distinguish the details of ground targets. In this paper, we tend to use point density to represent spatial resolution. For all products, the measuring point density of LiDAR is 5–10 points/m^2^, and that of MBES is 10–20 points/m^2^. Afterwards, a gridded digital elevation model (DEM) with a resolution of 1 m was generated by linearly interpolating the point cloud. According to the actual type of substrate in the survey area, three sample species were selected as sands, reefs, and rocks. The number of samples was set according to the area percentage of different substrates in the study area to make the spatial distribution of training samples more reasonable.

(3) Feature selection

Feature selection is helpful to avoid correlated features and improve classification accuracy. It has been proven in our previous research that, with fewer features mentioned in this paper, the classification accuracy would decline obviously, thus 16 features were chosen for experimental verification, eight LiDAR features and eight MBES features.

(4) Model performance

In our experiment, a five-fold cross-validation strategy was adopted and mean errors and accuracy metrics are presented to understand more about model performance. Among the nine classification combinations, the PSO-SVM classifier based on multifeatures had the highest overall accuracy of 96.71%, and its kappa coefficient was 0.94. Over-fitting and underfitting can occur in machine learning, in particular. The potential for overfitting depends not only on the number of parameters and data, but also on the conformability of the model structure with the data shape and the magnitude of model error compared to the expected level of noise or error in the data. In order to avoid over-fitting, the following solutions are given: (1) select appropriate stopping criteria to make the training of the machine to a suitable extent; (2) keep verification data sets and verify training results; and (3) get additional data for cross-validation.

(5) Practical importance

LiDAR bathymetry is limited by laser penetration and water turbidity. Its maximum bathymetric range is generally less than 50 m, so it is suitable for clearer shallow water areas. The attenuation of sound in water is much lower than that of laser, so MBES bathymetry is suitable for deeper areas. MBES transducers are usually mounted on ships, and it is difficult to approach shallow coastal areas due to the safety factors of sailing. LiDAR scanners are usually mounted on airplanes or unmanned aerial vehicles, and are almost unaffected by ground conditions. These two sources of data sets can be comprehensively utilized to achieve full coverage measurement of islands and reefs. To sum up, it was necessary to combine two data sources for our study, and we tend to believe that the method has a certain universality in similar areas.

## 6. Conclusions

This paper comprehensively considers the features of both airborne LiDAR waveforms and ship-borne MBES backscatter images and proposes a seafloor classification method using extracted multifeatures. Three kinds of classifiers, SVM, GA-SVM, and PSO-SVM, were used to classify the seafloor types around Yuanzhi Island in the South China Sea. In this survey area, three types of seafloor (sands, reefs, and rocks) were detected. The influence of different classifiers and different features on classification was obtained through quantitative analysis. In general, the classification results of the three classifiers achieved good performance. The overall accuracy and kappa coefficient of the PSO-SVM were higher (up to 96.71% and 0.94, respectively) than those of GA-SVM and SVM. The experimental results indicate that the fusion of LiDAR and MEBS data can reveal more features of seafloor, which is beneficial in improving the accuracy and reliability of seafloor classification. After merging the multifeatures, the classification accuracy of SVM, GA-SVM, and PSO-SVM increased by an average of 9.06%, 3.60%, and 2.75%, respectively.

In addition, although the multifeatures can be obtained through feature extraction, excessive features influence the efficiency of target detection and classification. If the correlation between features and seafloor types is small, then overfitting of the target detection and classification may occur, thereby decreasing the classification accuracy. In the future, feature selection criteria that are based on principal component analysis (PCA) or factor analysis (FA) will be used to select the most important feature parameters from multifeatures.

## Figures and Tables

**Table 1:** Expressions and explanations of light detection and ranging (LiDAR) features. FWHM, full width at half maximum.

Feature	Expression	Explanation
Amplitude	Amplitude	Characterize the return intensity as the maximum energy
Peak Location	Peak Location	Time when the return energy reaches the peak
FWHM	$\mathrm{FWHM}=2\sqrt{2ln2}\cdot W$	Width of the waveform when the return energy is half of its amplitude
Skewness	$\mathrm{Skewness}=\frac{1}{n}\overset{n}{\underset{i=1}{\sum }}\frac{{\left(x_{i}-\bar{x}\right)}^{3}}{{\left(\sqrt{\frac{1}{n}\sum_{i=1}^{n}{\left(x_{i}-\bar{x}\right)}^{2}}\right)}^{3}}$	Description of the distribution trend of waveform energy
Kurtosis	$\mathrm{Kurtosis}=\frac{1}{n}\overset{n}{\underset{i=1}{\sum }}\frac{{\left(x_{i}-\bar{x}\right)}^{4}}{{\left(\frac{1}{n}\sum_{i=1}^{n}{\left(x_{i}-\bar{x}\right)}^{2}\right)}^{2}}-3$	Characterization of the height of peaks
Area	$A=\overset{n}{\underset{i=1}{\sum }}a_{i}$	Characterization of the accumulation of all energy in waveforms
Distance	$\mathrm{Distance}=\frac{3\times 10^{8}\times \Delta t}{2\cdot \left(1+78.7\times \frac{P}{\left(273.15+T\right)}\times 10^{-6}\right)}$	Distance from the laser reference point to the target
Cross-section	$\sigma =C_{cal}\cdot R_{i}^{4}PW$	Action area between the seafloor and return signals

**Table 2:** Expressions and explanations of multibeam echo sounding (MBES) features. STD, standard deviation. ASM, angular second moment.

Feature	Expression	Explanation
Mean	$\mathrm{Mean}=\overset{N}{\underset{i=1}{\sum }}\overset{N}{\underset{j=1}{\sum }}P\left(i,j|d,\theta \right)*i$	Reflects the degree of texture rules
STD	$\mathrm{Std}=\sqrt{\overset{N}{\underset{i=1}{\sum }}\overset{N}{\underset{j=1}{\sum }}P\left(i,j|d,\theta \right)*{\left(i-Mean\right)}^{2}}$	Measurement of pixel value and mean deviation
Entropy	$\mathrm{Entropy}=-\overset{N}{\underset{i=1}{\sum }}\overset{N}{\underset{j=1}{\sum }}P\left(i,j|d,\theta \right)*InP\left(i,j|d,\theta \right)$	Measurement of gray image information
Homogeneity	$\mathrm{Homogeneity}=\overset{N}{\underset{i=1}{\sum }}\overset{N}{\underset{j=1}{\sum }}P\left(i,j|d,\theta \right)*\frac{1}{1+{\left(i-j\right)}^{2}}$	Measurement of local gray homogeneity in the image
Contrast	$\mathrm{Contrast}=\overset{N}{\underset{i=1}{\sum }}\overset{N}{\underset{j=1}{\sum }}P\left(i,j|d,\theta \right)*{\left(i-j\right)}^{2}$	Reflects the total amount of local gray changes in the image
ASM	$\mathrm{ASM}=\overset{N}{\underset{i=1}{\sum }}\overset{N}{\underset{j=1}{\sum }}P{\left(i,j|d,\theta \right)}^{2}$	Measurement of gray distribution homogeneity in the image
Correlation	$\mathrm{Correlation}=\overset{N}{\underset{i=1}{\sum }}\overset{N}{\underset{j=1}{\sum }}\frac{\left(i-Mean\right)*\left(j-Mean\right)*P{\left(i,j|d,\theta \right)}^{2}}{Variance}$	Measurement of gray linear relation
Dissimilarity	$\mathrm{Dissimilarity}=\overset{N}{\underset{i=1}{\sum }}\overset{N}{\underset{j=1}{\sum }}P\left(i,j|d,\theta \right)*\left|i-j\right|$	Similar to contrast but linearly increasing

**Table 3:** Evaluation of classification results based on LiDAR waveform features.

Model No.	Algorithm	Classes	Predictions			PA (%)	OA (%) (STD)	Kappa (STD)
			Reefs	Sands	Rocks			
Model 1	SVM	reefs	492	4	22	94.98	88.73 (1.70)	0.79 (0.02)
		sands	4	1511	45	96.86		
		rocks	168	48	288	57.14		
		UA (%)	74.10	96.67	81.13	-		
Model 2	GA-SVM	reefs	493	1	24	95.17	92.10 (1.25)	0.86 (0.01)
		sands	2	1518	40	97.31		
		rocks	130	7	367	72.82		
		UA (%)	78.88	99.48	85.15	-		
Model 3	PSO-SVM	reefs	495	1	22	95.56	94.54 (0.94)	0.90 (0.01)
		sands	1	1526	33	97.82		
		rocks	79	5	420	83.33		
		UA (%)	86.09	99.61	88.42	-		

**Table 4:** Evaluation of classification results based on MBES backscatter features.

Model No.	Algorithm	Classes	Predictions			PA (%)	OA (%) (STD)	Kappa (STD)
			Reefs	Sands	Rocks			
Model 1	SVM	reefs	383	111	24	73.94	78.82 (1.33)	0.61 (0.01)
		sands	161	1325	74	84.94		
		rocks	7	170	327	64.88		
		UA (%)	69.51	82.50	76.94	-		
Model 2	GA-SVM	reefs	492	13	13	94.98	90.32 (1.12)	0.83 (0.01)
		sands	150	1392	18	89.23		
		rocks	5	51	448	88.89		
		UA (%)	76.04	95.60	93.53	-		
Model 3	PSO-SVM	reefs	510	4	4	98.46	93.38 (0.81)	0.88 (0.01)
		sands	106	1433	21	91.86		
		rocks	2	34	468	92.86		
		UA (%)	82.52	97.42	94.93	-		

**Table 5:** Evaluation of classification results based on multifeatures.

Model No.	Algorithm	Classes	Predictions			PA (%)	OA (%) (STD)	Kappa (STD)
			Reefs	Sands	Rocks			
Model 1	SVM	reefs	469	3	46	90.54	92.84 (1.21)	0.87 (0.01)
		sands	10	1540	10	98.72		
		rocks	91	25	388	76.98		
		UA (%)	82.28	98.21	87.39	-		
Model 2	GA-SVM	reefs	483	2	33	93.24	94.81 (1.02)	90.65 (0.01)
		sands	7	1550	3	99.36		
		rocks	70	19	415	82.34		
		UA (%)	86.25	98.66	92.02	-		
Model 3	PSO-SVM	reefs	490	1	27	94.60	96.71 (0.56)	0.94 (0.01)
		sands	2	1549	9	99.30		
		rocks	42	4	458	90.87		
		UA (%)	91.76	99.68	92.71	-		

**Table 6:** Model evaluation results based on five-fold cross validation. STD, standard deviation.

Model	OA (%)	SVM	GA-SVM	PSO-SVM
LiDAR	A	90.82	93.79	94.28
	B	88.81	90.48	93.79
	C	87.85	91.89	93.64
	D	86.43	92.76	95.12
	E	89.76	91.58	95.87
	Average	88.73	92.10	94.54
	STD	1.70	1.25	0.94
MBES	A	78.41	89.64	92.46
	B	77.23	88.92	93.45
	C	79.42	90.45	93.94
	D	80.74	91.88	92.68
	E	78.28	90.71	94.35
	Average	78.82	90.32	93.38
	STD	1.33	1.12	0.81
Multifeatures	A	93.41	95.45	96.74
	B	91.52	93.41	95.87
	C	92.04	94.15	96.65
	D	92.62	95.14	96.86
	E	94.59	95.91	97.43
	Average	92.84	94.81	96.71
	STD	1.21	1.02	0.56
